# H_2_S Protects from Rotenone-Induced Ferroptosis by Stabilizing Fe-S Clusters in Rat Cardiac Cells

**DOI:** 10.3390/cells13050371

**Published:** 2024-02-21

**Authors:** Sara Linjacki, Yuehong Wang, Navjeet Baath, Devin Mantle, Guangdong Yang

**Affiliations:** 1 School of Natural Sciences, Laurentian University, Sudbury, ON P3E 2C6, Canada; saralinjacki@gmail.com (S.L.); wangyh0327@126.com (Y.W.); navjeet.baath@medportal.ca (N.B.); dmantle@laurentian.ca (D.M.); 2 Cardiovascular and Metabolic Research Unit, Laurentian University, Sudbury, ON P3E 2C6, Canada

**Keywords:** H_2_S, rotenone, cardiac cells, Fe-S clusters, ferroptosis

## Abstract

Hydrogen sulfide (H_2_S) has been recently recognized as an important gasotransmitter with cardioprotections, and iron is vital for various cellular activities. This study explored the regulatory role of H_2_S on iron metabolism and mitochondrial functions in cultured rat cardiac cells. Rotenone, a mitochondrial complex I inhibitor, was used for establishing an in vitro model of ischemic cell damage. It was first found that rotenone induced oxidative stress and lipid peroxidation and decreased mitochondrial membrane potential and ATP generation, eventually causing cell death. The supplement of H_2_S at a physiologically relevant concentration protected from rotenone-induced ferroptotic cell death by reducing oxidative stress and mitochondrial damage, maintaining GPx4 expression and intracellular iron level. Deferiprone, an iron chelator, would also protect from rotenone-induced ferroptosis. Further studies demonstrated that H_2_S inhibited ABCB8-mediated iron efflux from mitochondria to cytosol and promoted NFS1-mediated Fe-S cluster biogenesis. It is also found that rotenone stimulated iron-dependent H_2_S generation. These results indicate that H_2_S would protect cardiac cells from ischemic damage through preserving mitochondrial functions and intracellular Fe-S cluster homeostasis.

## 1. Introduction

Hydrogen sulfide (H_2_S) is traditionally known as a toxic gas and environmental pollutant, but recently considerable evidence has indicated that H_2_S at a very small amount plays a crucial role in physiological activities in the human body [1,2,3]. H_2_S is now recognized as the third known endogenous gaseous transmitter following nitric oxide and carbon monoxide [4,5], and can be endogenously generated from sulfur-containing compounds via enzymatic and non-enzymatic pathways [4,6,7]. Three enzymes have been discovered for generating H_2_S from cysteine: cystathionine gamma-lyase (CSE), cystathionine beta-synthase (CBS), and 3-mercaptopyruvate sulfurtransferase (3MST) [1,4]. The expressions of these genes are tissue specific. In cardiovascular systems, only CSE and 3MST are expressed [1,8,9]. The knockout of *CSE* diminished a majority of H_2_S production in the heart, while cardiac H_2_S levels were not altered in *3MST* deficient mice, suggesting that CSE acts as a major H_2_S-generating enzyme in cardiac cells [1,5]. Disrupted CSE expression and H_2_S generation have been found in a variety of vascular diseases, and enhanced CSE/H_2_S signal would be able to improve vascular functions and prevent vascular disorders [10,11,12,13]. Another important source of H_2_S is through non-enzymatic production from sulfur-containing molecules such as cysteine, thiosulfate, and persulfides [6,10,14]. Recent research found that iron would be able to chemically react with cysteine in the presence of pyridoxal-5′-phosphate (P5P) to release H_2_S [6]. S-sulfhydration is a newly discovered mechanism for H_2_S signaling, which can modify specific cysteine residues in target proteins for inducing the changes of protein activity [5,15,16]. A plethora of literature over the past few decades has demonstrated that H_2_S plays significant protective roles in the cardiovascular system via the actions of inflammation inhibition, antioxidant regulation, vasodilation, and energy metabolism, etc. [1,2,10,11,12,13]. All these findings suggest that H_2_S can be a target for drug design to prevent and cure cardiovascular diseases.

Iron is an essential nutritional trace element and is crucial for human life. Iron participates in various metabolic processes inside the cells [17,18], and is mostly used in mitochondria for the synthesis of iron-sulfur (Fe-S) clusters and heme proteins [19,20]. Fe-S clusters are essential components of the electron transport chain (ETC) in the mitochondria, especially in complex I, where both iron and sulfur can accept and donate their electrons to facilitate ATP generation [20,21]. Heme is an important co-factor for many proteins involved in oxygen transport and enzymatic activity [22]. Maintenance of iron homeostasis is critical to normal cell functions. A variety of evidence suggest that the interruption of cellular iron homeostasis would lead to many cardiovascular diseases, especially ischemic heart diseases caused by myocardial ischemia-reperfusion (I/R) injury [18,20]. Higher levels of labile iron would induce oxidative stress, leading to mitochondrial damage, lipid peroxidation, and eventually ferroptosis [23,24]. Rotenone, a broad-spectrum pesticide, is a second-class hazardous agent for humans and is often used by researchers to mimic ischemic cell damage [25,26,27]. Many studies have shown that rotenone inhibits complex I in the mitochondrial ETC, leading to the disruption of ATP generation and initiation of cell death [25,28,29]. The contribution of mitochondrial dysfunction to cardiovascular disease pathogenesis is well recognized. However, the regulatory roles of H_2_S in iron metabolism, Fe-S cluster stability, and the mitochondrial function of cardiomyocytes are not fully understood.

In this study, by using an in vitro model of mitochondrial dysfunctions in rat cardiomyocytes (H9C2) with the mitochondrial respiratory complex I inhibitor rotenone, we found that H_2_S would be able to block rotenone-induced ferroptosis by inhibiting the accumulations of labile iron and lipid peroxides via the stabilization of Fe-S cluster-containing proteins. These results indicate that H_2_S is a promising agent for protecting ischemic heart damage through preserving mitochondrial functions and intracellular Fe-S cluster homeostasis.

## 2. Materials and Methods

### 2.1. Cell Culture

H9C2 rat cardiomyocytes were purchased from ATCC (Manassas, VA, USA, CRL-1446TM). The cells were grown in a humidified CO_2_ incubator at 37 °C with Dulbecco’s modified Eagle’s medium (Sigma-Aldrich, St. Louis, MO, USA) containing 10% heat-inactivated fetal bovine serum, 1% streptomycin/penicillin.

### 2.2. Cell Viability

Cell viability was detected by MTT assay in 96-well plates as previously described [7,30]. The absorbance for the solubilized formazan crystals were measured with a FLUOstar OPTIMA microplate reader (BMG Labtech Inc., Ortenberg, Germany) at a wavelength of 570 nm.

### 2.3. DAPI Staining

DAPI staining was performed to detect the changes in condensed nuclei from apoptotic cells. After different treatments, the cells were fixed in 3.7% formaldehyde for 15 min. Subsequently, the cells were washed thrice in PBS and incubated with DAPI labeling solution (Thermo Fisher Scientific, Toronto, ON, Canada) for 5 min in the dark followed by three times of wash with PBS. The changes in nuclear morphology were observed with EVOS M5000 fluorescent microscope (Thermo Fisher Scientific). The percentage of apoptosis was calculated based on the ratio of cell number with condensed nucleus to the total cell number counted.

### 2.4. Detection of ROS, Malondialdehyde (MDA), and Mitochondrial Membrane Potential

Intracellular reactive oxygen species (ROS) levels were detected with 2,7-dichlorodihydrofluorescein diacetate (H2DCFDA) dye (Thermo Fisher Scientific) [30]. ROS level was normalized to cell number and expressed as a percentage of the control cells. Lipid peroxidation was determined by measuring the contents of MDA with an assay kit from Abcam (Toronto, ON, Canada) [30]. MDA levels were normalized to the total protein amounts and expressed as fold change to the control. Mitochondrial membrane potential was assessed with a JC1 dye (Abcam) and expressed as the ratio of red–green fluorescence intensity [30].

### 2.5. Western Blotting

The total proteins were extracted in a radioimmunoprecipitation assay buffer containing protease inhibitor (Sigma-Aldrich), and then separated on a 10% SDS-PAGE gel followed by transferring to a PVDF membrane (Pall Corporation, Pensacola, FL, USA) [16]. After blocking, the membranes were incubated with appropriate primary antibodies and peroxidase-conjugated secondary antibodies. The following antibodies were diluted as follows: CSE (1:1000, Abnova, Taipei, Taiwan), cleaved caspase 3 (1:1000, Cell Signaling Technology, Danvers, MA, USA), GPx4 (1:500, Santa Cruz Biotechnology Inc., Santa Cruz, CA, USA), ACSL4 (1:500, Santa Cruz Biotechnology Inc.), Alox12 (1:500, Santa Cruz Biotechnology Inc.), cysteine desulfurase (NFS1, 1:1000, Abnova), and GAPDH (1:500, Sigma-Aldrich).

### 2.6. S-Sulfhydration Assay

NFS1 S-sulfhydration was detected with modified biotin switch assay as described previously [16]. An anti-NFS1 antibody was used for western blotting analysis of biotinylated proteins. NFS1 S-sulfhydration was normalized to total NFS1 protein (“input”).

### 2.7. Real-Time PCR

Total RNAs were isolated using TriReagent (Sigma-Aldrich) and reverse-transcribed to cDNA using Verso cDNA synthesis kit (Thermo Fisher Scientific) as previously described [7]. The sequences of primers were used as follow: *ABCB7* (5′-AGTCGCAAAATTGGCTGGTCTTC-3′ and 5′-CTGTATGCGGGTGCTCTGTGTATG-3′), *ABCB8* (5′-CGCCAAGTACACGAGGGAGCAT-3′ and 5′-GTAAGGCGCGGGGACAGAATAGA-3′), *mitoferrin 1* (5′-GGAGCGATGGCCGGGATTCTGG-3′ and 5′-CCGGATACAACTGAGGGCTGACTG-3′), *mitoferrin 2* (5′-TCGGGCAGTGTGGCAAAATGAAG-3′ and 5′-CTCTGGCCTGTACCCCTCGGAAGT-3′), *IRP1* (5′-GCCCTGCCGCTCGCTACTT-3′ and 5′-GTTTCGCCGGGGAGATACT-3′), *IRP2* (5′-TCCCCGCCCGTGTTGTTC-3′ and 5′-ACGATCCCCGGCAGGTAGTCT-3′), *ferroportin* (FPN, 5′-AGTCATTGGCTGTGGTTTCATTTC-3′ and 5′-AAGGCCACAGTTCCCATTATTCC-3′), *TFR* (5′-ACCATACTCAGTTTCCGCCATCTC-3′ and 5′-GGTACCCCTCCAGCCACTCAG-3′), *ferritin* (5′ACGGGGYGAGTGGAGATGATTT-3′ and 5′-ATATAGCCGACGCAAGGGAAGC-3′), *GAPDH* (5′-GGGGCCAAAAGGGTCATCATCTCC-3′ and 5′-GCCGCCTGCTTCACCACCTTCTT-3′).

### 2.8. Measurement of Labile Iron Levels

Labile iron contents in mitochondrial and cytosolic fractions were analyzed with a modified ferrozine colorimetric assay [7]. After treatments, the cells were collected for extraction of cytosol and mitochondria fractions with a cytosol/mitochondria fractionation kit from Thermo Fisher Scientific. Iron concentrations were expressed as nmole of iron per mg of protein.

### 2.9. ACO Activity

Aconitase (ACO) activity was determined with an aconitase assay kit from Abcam as described previously [7]. The activity of ACO was expressed as the fold change to the control cells after normalization to the amount of total proteins.

### 2.10. ATP Generation

ATP generation was measured using an ATP bioluminescent assay kit according to manufacturer instructions [14]. Briefly, after different treatments, the cells were collected and lysed. The supernatants were mixed with an ATP assay reagent containing luciferin and luciferase for 5 min at dark. The luminescence was then measured with a FLUOstar OPTIMA microplate spectrophotometer. ATP contents were calculated based on ATP calibration curve and expressed as a percentage of the control group after normalization to the amount of total proteins.

### 2.11. Complex I Activity

Complex I activity was measured using a mitochondrial complex I activity colorimetric assay kit (Thermo Fisher Scientific) according to the manufacturer instructions. Complex I activity was expressed as fold change to the control groups after normalization to the amount of total proteins [28].

### 2.12. Mitochondrial Oxygen Consumption Rate Assay

The mitochondrial oxygen consumption rate was analyzed with a mitochondrial stress test kit (Abcam) and expressed as a percentage of the control cells after determining the changes in fluorescence signal over 30 min [31].

### 2.13. Measurement of CSE Activity and H_2_S Production

CSE activity was measured with a colorimetric assay for the determination of pyruvate formation and expressed as the optical density (OD) of absorbance at 720 nm per mg of protein [32,33]. H_2_S generation was detected with a lead acetate (PbAc) paper method as previously described [6,7]. The amount of H_2_S released was expressed as a percentage of the control group.

### 2.14. GPx4 Knockdown by Small Interfering RNA (siRNA)

GPx4 siRNA and negative siRNA were purchased from Thermo Fisher Scientific. H9C2 cells were transfected with either GPx4 siRNA (100 nM) or negative siRNA (100 nM) for 24 h using the Lipofectamine^TM^ 2000 transfection agent (Invitrogen, Carlsbad, CA, USA) followed by incubation with rotenone (10 µM) for an additional 24 h.

### 2.15. NFS1 Overexpression

The NFS1 cDNA vector was purchased from Addgene (Watertown, MA, USA) [34]. H9C2 cells were transfected with 1 µg NFS1 cDNA vector for 48 h using the Lipofectamine^TM^ 2000 transfection agent (Invitrogen).

### 2.16. Statistical Analysis

All experiments were performed independently at least three times. Statistical comparisons were made using Student’s *t*-tests or one-way ANOVA with a Tukey’s post hoc analysis, where applicable. The data were presented as means ± standard error (SE). *p*-value < 0.05 was considered statistically significant.

## 3. Results

### 3.1. H_2_S Protected Cardiac Cells from Rotenone-Induced Ferroptosis

**Figure 1:**
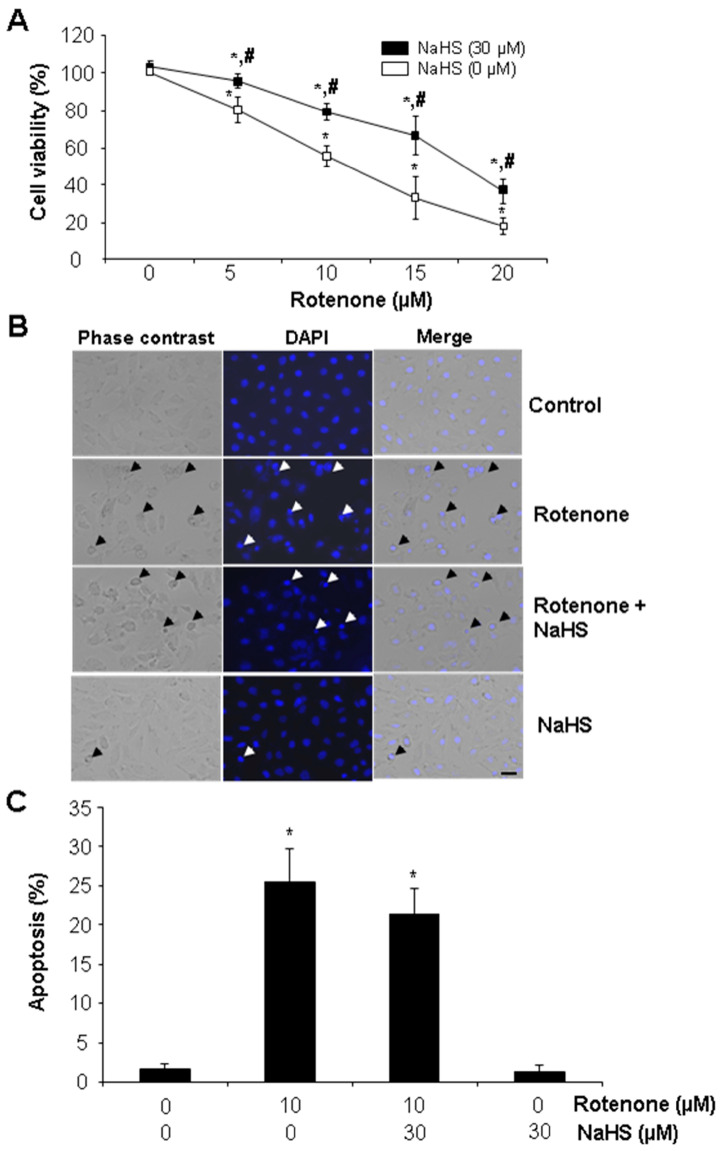
H_2_S protected rotenone-induced cell death. (**A**) H_2_S reversed rotenone-inhibited cell viability. After cells were incubated with rotenone at different concentrations (0–20 µM) with or without 30 µM NaHS for 24 h, the cell viability was detected by MTT. * *p* < 0.05 versus the control cells; # *p* < 0.05 versus the cells treated with rotenone only. The experiments were repeated three times. (**B**,**C**) H_2_S had no effect on rotenone-induced apoptosis. After cells were incubated with 10 µM rotenone with or without 30 µM NaHS for 24 h, apoptosis was detected by staining cells with DAPI. Apoptosis was calculated based on the ratio of nucleus-condensed cells to total cells counted. Arrow points to nucleus-condensed cells. Scale bar in (**B**): 20 µm. More than 600 cells were counted in each group. * *p* < 0.05 versus control cells.

The effects of rotenone and NaHS on cell viability of cultured rat cardiac cells (H9C2) was first tested with MTT assay. Rotenone inhibited cell viability in a dose-dependent manner. The cell viability in 10 µM rotenone-treated cells was only 60% of the control cells. Rotenone at 20 µM inhibited cell viability by 80% (Figure 1A). Exogenously applied H_2_S at physiological relevant concentration (30 µM) significantly restored rotenone-reduced cell viability at all the tested doses (Figure 1A). Next, DAPI was used to stain the cells for observing nucleus-condensed apoptotic cells. As shown in Figure 1B,C, rotenone at 10 µM induced a higher rate (25%) of apoptosis, which was not changed by co-incubation with 30 µM NaHS. NaHS alone had no effect on apoptosis. These data suggest that H_2_S-protected cell death by rotenone may be independent of apoptosis.

**Figure 2:**
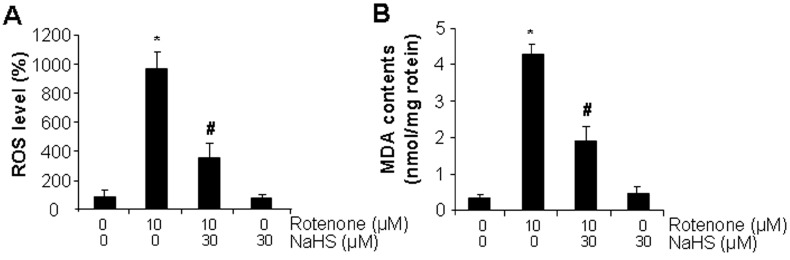

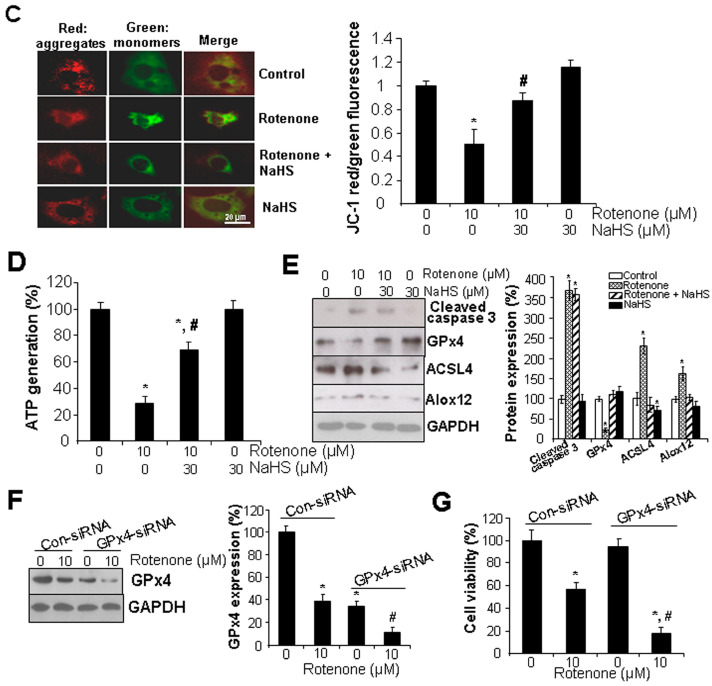
H_2_S reversed rotenone-induced ferroptosis. In (**A**–**E**), cells were incubated with 10 µM rotenone in the presence or absence of 30 µM NaHS for 24 h. Cells were then subjected for (**A**) ROS measurement with a fluorescent probe H2DCFDA, (**B**) lipid peroxidation by measuring the contents of MDA, (**C**) mitochondrial membrane potential change with JC-1 staining, (**D**) ATP generation with an ATP bioluminescent assay kit, (**E**) and protein expressions by Western blotting. *, *p* < 0.05 versus the control cells; #, *p* < 0.05 versus the cells treated with rotenone only. The experiments were repeated four times. In (**F**,**G**) the cells were transected with GPx4-siRNA for 24 h followed by incubation with 10 µM rotenone for an additional 24 h, (**F**) GPx4 expression and (**G**) cell viability were then measured. *, *p* < 0.05 versus control siRNA (con-siRNA)-transfected cells without rotenone treatment; #, *p* < 0.05 versus GPx4 siRNA-transfected cells without rotenone treatment. Experiments were repeated three times.

**Figure 3:**
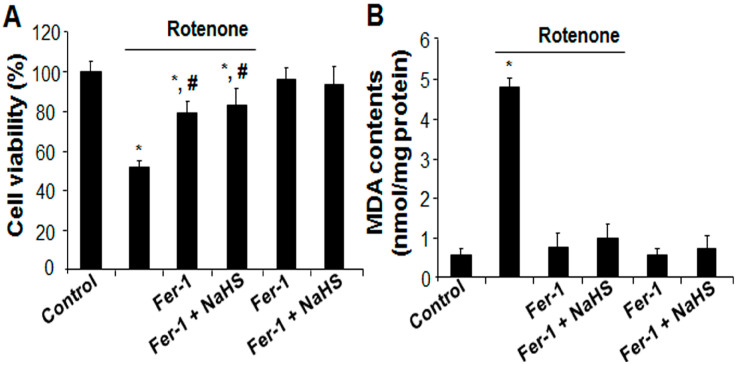

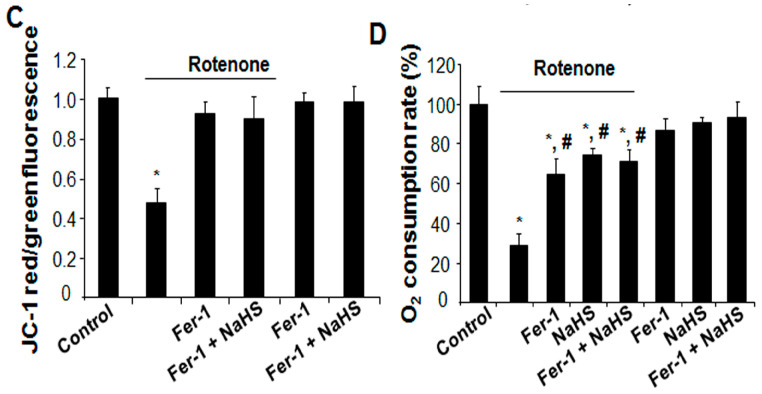
Fer-1 inhibited rotenone-induced ferroptosis. After cells were incubated with 10 µM rotenone in the presence or absence of 10 µM Fer-1 and/or 30 µM NaHS for 24 h, cells were subjected to (**A**) cell viability assay with MTT, (**B**) lipid peroxidation by measuring the contents of MDA, (**C**) mitochondrial membrane potential change with JC-1 staining, and (**D**) oxygen (O_2_) consumption rate with a mitochondrial stress test kit. * *p* < 0.05 versus control cells; # *p* < 0.05 versus cells treated with rotenone only. Experiments were repeated four times.

It was further found that rotenone markedly induced higher ROS levels (Figure 2A) and MDA content (Figure 2B), which would be inhibited partially and significantly by NaHS. The mitochondrial membrane potential (ΔΨm) was also examined with JC-1 dye. As observed in Figure 2C, in comparison to the control group, rotenone robustly decreased the intensity ratio of red–green fluorescence, which could be restored by the addition of NaHS. There was no difference between the control and NaHS treatment group. These results indicate that H_2_S protects cardiac cells from rotenone-induced mitochondrial damage. In line with this, ATP generation was dramatically lower in rotenone-treated cells but significantly higher in rotenone and H_2_S co-incubated cells (Figure 2D). GPx4, a key enzyme in protecting lipid peroxidation and ferroptosis [18,30], was lower in rotenone-treated cells (Figure 2E). In contrast, the expressions of ACSL4 and Alox12, two ferroptosis marker proteins involved in phospholipid peroxidation [30,35,36], were significantly higher in rotenone-treated cells. NaHS co-incubation would markedly restored rotenone-altered expressions of GPx4, ACSL4, and Alox12. Caspase 3 is essential for the initiation and execution of apoptosis [37]. Figure 2E showed that rotenone activated cleaved caspase 3, which was not altered by the addition of NaHS. To further explore the contribution of GPx4 in rotenone-induced cell death, siRNA was used to knockdown GPx4 (Figure 2F). In comparison with the control cells transfected with negative siRNA, rotenone inhibited cell viability by more than 64% in GPx4-siRNA transfected cells, pointing to the protective role of GPx4 against rotenone-caused cell death (Figure 2G). Interestingly, Fer-1, a selective ferroptosis inhibitor, would also be able to decrease rotenone-induced cell death (Figure 3A), lipid peroxidation (Figure 3B), and mitochondrial damage (Figure 3C). In supporting these data, Figure 3D showed that rotenone significantly inhibited oxygen consumption rate, which would be prevented by the supplement of either Fer-1 or NaHS. Fer-1. H_2_S did not show additive effect in inhibiting rotenone-induced mitochondrial dysfunctions (Figure 3A–D). In combination, these data suggest that H_2_S protects cardiac cells from rotenone-induced ferroptosis but not apoptosis.

### 3.2. H_2_S Improved Rotenone-Induced Iron Metabolism Dysfunctions

**Figure 4:**
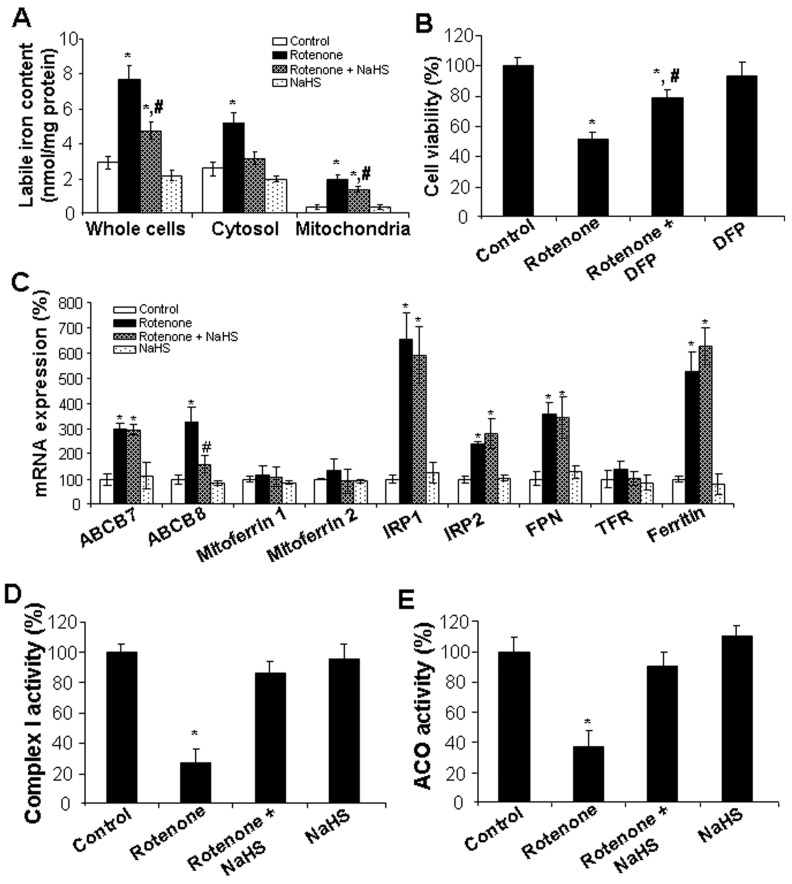
H_2_S attenuated rotenone-induced labile iron level. Fer-1 inhibited rotenone-induced ferroptosis. After cells were incubated with 10 µM rotenone in the presence or absence of 30 µM NaHS or 100 µM DFP for 24 h, they were subjected for (**A**) labile iron measurement with ferrozine colorimetric assay, (**B**) cell viability assay with MTT, (**C**) mRNA expression by real-time PCR, (**D**) measurements of mitochondrial complex I activity, and (**E**) ACO activity. * *p* < 0.05 versus control cells; # *p* < 0.05 versus cells treated with rotenone only. Experiments were repeated four times.

Ferroptosis is a regulated cell death driven by iron-dependent lipid peroxidation [18,38]. To further investigate how H_2_S protects from rotenone-induced ferroptosis, labile iron levels were measured with a ferrozine colorimetric assay. Figure 4A showed that labile iron level in whole cell lysates from rotenone-treated cells was about 2.6 times that in control cells, which would be significantly reduced by NaHS co-incubation. Further analysis of cytosolic and mitochondrial fractions demonstrated that rotenone induced more labile iron level in mitochondria (6.7 times of the control) than that in cytosol (1.9 times of the control). In comparison to the cells treated with rotenone, NaHS completely brought labile iron to the same level as control cells in cytosol, but only partially attenuated labile iron level in mitochondria (Figure 4A). Deferiprone (DFP), an iron chelator, would also significantly attenuate rotenone-inhibited cell viability by 40% (Figure 4B), pointing to the possible mediation of iron overload in rotenone-induced ferroptosis. To explore the mechanisms underlying the induction of labile iron level by rotenone, several iron metabolism-related genes were analyzed by real-time PCR. As shown in Figure 4C, the mRNA expressions of *ABCB7*, *ABCB8*, *IRP1*, *IRP2*, *FPN*, and *ferritin* in rotenone-incubated cells were significantly higher than those in the control cells. The co-incubation with NaHS only inhibited rotenone-induced *ABCB8*, but no other genes. In addition, the mRNA levels of *mitoferrin 1*, *mitoferrin 2*, and *TFR* were not affected by either rotenone and/or NaHS. Mitochondrial complex I activity has the largest number of iron-sulfur (Fe-S) cluster-containing proteins in the electron transport chain, which is known to be inhibited by rotenone [26]. ACO contains a [4Fe-4S]-cluster and is an essential enzyme in the citric acid cycle [7]. We further observed that rotenone markedly inhibited the activities of mitochondrial complex I (Figure 4D) and ACO (Figure 4E), both of which could be decreased by NaHS co-incubation. These data suggest that H_2_S may improve cellular iron balance by maintaining the stability of Fe-S cluster.

### 3.3. NFS1 Overexpression Enhanced the Protective Role of H_2_S against Rotenone-Induced Cell Death and Labile Iron Level

**Figure 5:**
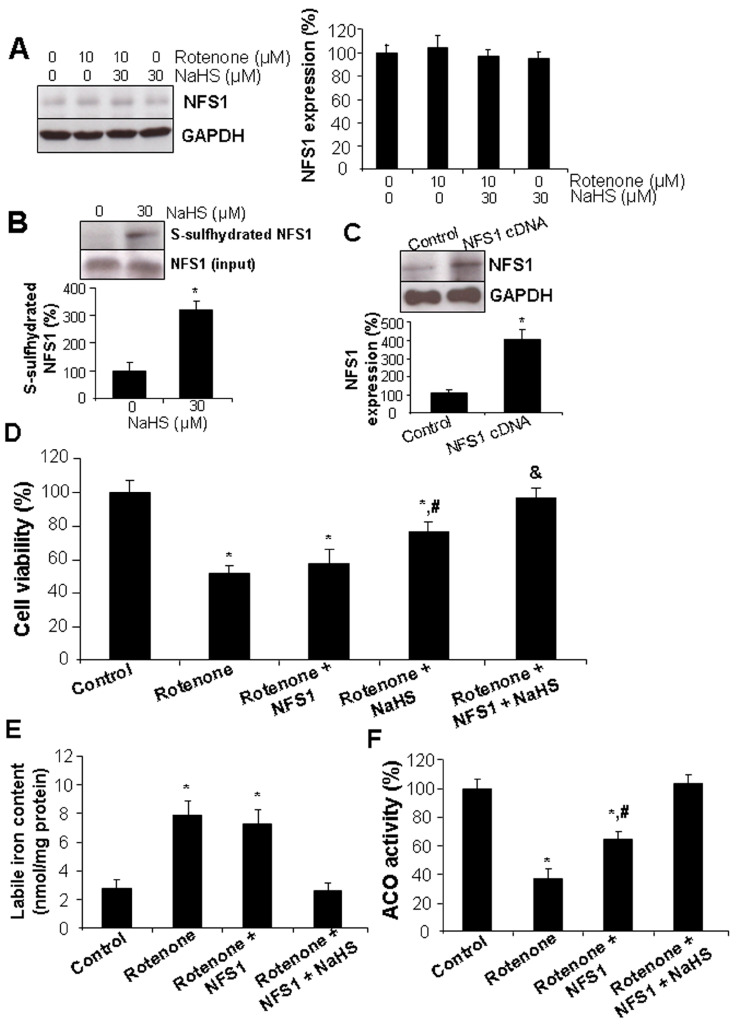
NFS1 overexpression enhanced the protective role of H_2_S against rotenone-induced cell death and labile iron level. (**A**) NFS1 was not affected by either rotenone or H_2_S. After cells were incubated with 10 µM rotenone in the presence or absence of 30 µM NaHS for 24 h, they were collected for Western blotting analysis of NFS1 expression. (**B**) NFS1 was S-sulfhydrated by H_2_S. After cells were incubated with 30 µM NaHS for 24 h, they were collected for biotin switch detection of NFS1 S-sulfhydration. *, *p* < 0.05 versus control cells. In (**C**–**F**), cells were transfected with NSF1 cDNA in the presence or absence of 10 µM rotenone and/or 30 µM NaHS for 24 h. Cells were then subjected to (**C**) NFS1 protein expression by Western blotting, (**D**) cell viability by MTT assay, (**E**) labile iron measurement with ferrozine colorimetric assay, and (**F**) measurement of ACO activity. * *p* < 0.05 versus control cells; # *p* < 0.05 versus cells treated with rotenone only. Experiments were repeated three times.

NFS1 is a central enzyme in mitochondria for the biosynthesis and stability of Fe-S clusters [34,39]. It was first found that neither rotenone nor H_2_S treatment had an effect on NFS1 expression (Figure 5A). NSF1 was S-sulfhydrated the basal level and could be further enhanced in the presence of exogenously applied H_2_S (Figure 5B). To study the contribution of NFS1 in H_2_S-inhibited labile iron level and ferroptosis induced by rotenone, NFS1 was overexpressed in H9C2 cells (Figure 5C). NFS1 overexpression had no effect on rotenone-inhibited cell viability, however, NFS1 would be able to significantly enhance the protective role of H_2_S against rotenone-induced cell death (Figure 5D). Similarly, NFS1 overexpression would only inhibit the rotenone-induced labile iron level in the presence of H_2_S (Figure 5E). In addition, rotenone-reduced ACO activity could be partially restored by NFS1 overexpression and completely recovered to the basal level as in the control cells in the presence of both NFS1 overexpression and H_2_S treatment (Figure 5F). These results indicate that NFS1 contributes to the protective roles of H_2_S against rotenone-induced labile iron level and ferroptotic cell death by possibly stimulating Fe-S cluster biogenesis.

### 3.4. Rotenone Stimulated H_2_S Generation Independent of CSE Expression

**Figure 6:**
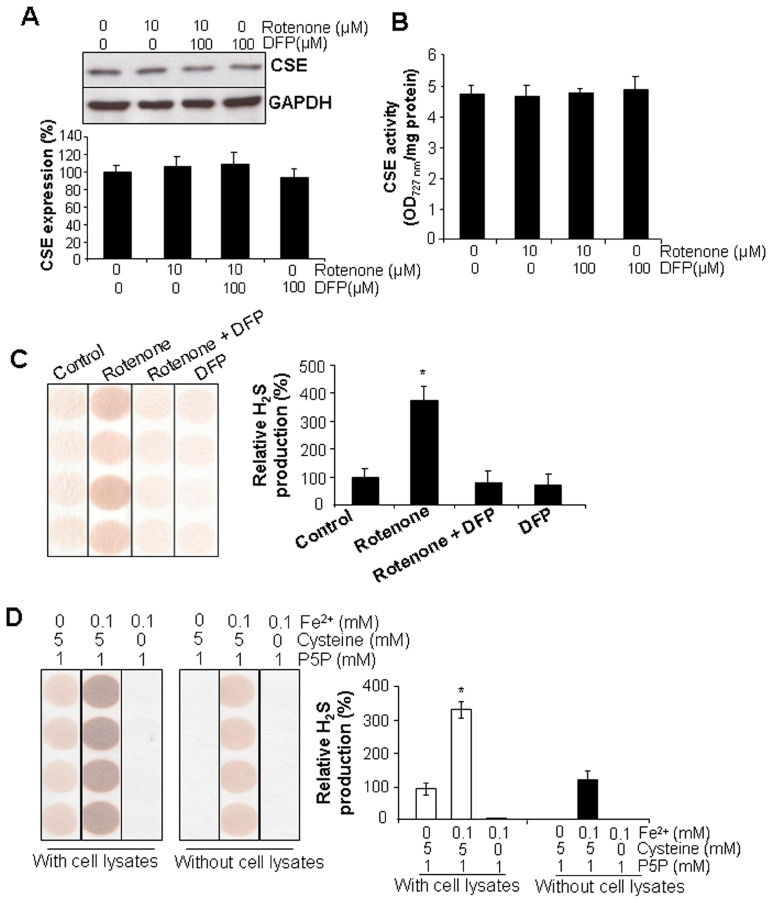
Rotenone stimulated H_2_S generation in an iron-dependent manner. (**A**,**B**) Rotenone and DFP had no effect on CSE protein expression and activity. After incubation with rotenone (10 μM) and/or DFP (100 μM) for 24 h, cells were collected for detection of CSE protein expression by Western blotting and CSE activity by the determination of pyruvate formation. (**C**) DFP reversed rotenone-induced H_2_S generation. After incubation with rotenone (10 μM) and/or DFP (100 μM) for 24 h, cells were collected for detection of H_2_S generation by lead acetate paper and then quantified by ImageJ software (Version 1.43). * *p* < 0.05 versus all other groups. (**D**) Iron induced H_2_S generation from cysteine in the absence of cell lysates. Iron (FeCl_2_) was mixed with cysteine and P5P in the presence or absence of cell lysates for H_2_S generation. * *p* < 0.05 versus all other groups. Experiments were repeated four times.

To further explore the mutual interaction of rotenone and H_2_S signaling, we detected the change of CSE/H_2_S system in H9C2 cells in response to rotenone treatment. CSE protein level and activity were not changed by rotenone with or without DFP (Figure 6A,B). In contrast, rotenone significantly induced H_2_S generation, and chelating iron with DFP inhibited rotenone-induced H_2_S generation (Figure 6C). Furthermore, in the absence of cell lysates, Fe^2+^ (100 µM) would be able to liberate H_2_S release from cysteine (Figure 6D). These data suggest that rotenone may stimulate H_2_S generation in an iron-dependent manner, independent of CSE enzyme.

## 4. Discussion

Ischemic heart disease is the leading cause of death worldwide, which occurs when blood flow and oxygen supply to the heart are restricted or reduced, eventually resulting in a heart attack and heart failure [40]. Cardiomyocytes are highly susceptible to mitochondrial dysfunctions and cell death induced by myocardial ischemia and reperfusion [12,41,42]. H_2_S is a well-known endogenous gaseous transmitter and participates in many physiological activities in our bodies [1,2,10]. The present study investigated the protective role of H_2_S on rotenone-induced cardiotoxicity and mitochondrial dysfunction. It is revealed that H_2_S improves cell survival by inhibiting labile iron accumulation and ferroptotic cell death caused by rotenone. Mechanistically, H_2_S maintains Fe-S cluster stability and complex I activity, improving mitochondrial functions and energy production.

Complex I is the first and largest multimeric enzyme complex of the mitochondrial respiratory chain, which is essential for electron transport and ATP production [28,43]. Disruption of complex I is often observed at the onset and during the development of ischemic heart disease, causing ATP depletion and excessive ROS generation [20]. To explore the protective roles of H_2_S against ischemia-induced cardiac cell death and functionally characterize the underlying molecular mechanisms, we used H9C2 cells as an in vitro model treated with complex I inhibitor rotenone. Emerging evidence has indicated that rotenone induces cell death by inhibiting complex I activity and causing mitochondrial damage [26,44]. With a MTT assay, this present study found that rotenone dose-dependently inhibited cell viability. However, with the addition of NaHS, rotenone-inhibited cell viability could be significantly reduced. In line with our findings, it has been widely shown that H_2_S protects cardiac cell death induced by various stressors [13,45,46]. Apoptosis significantly contributes to myocyte cell death in ischemic heart disease [41,42]. Although rotenone induced a higher level of apoptosis, H_2_S had no effect on rotenone-induced apoptotic cell death as evidenced by no change of condensed cell nuclei and cleaved caspase 3, suggesting that H_2_S may protect cell death in forms other than apoptosis. Accumulated evidence has shown that ferroptosis also plays a vital role in the initiation and development of many cardiovascular diseases [18,24]. We further provided several lines of evidence that H_2_S can protect rotenone-induced ferroptotic cell death. First, H_2_S markedly lowered rotenone-induced ROS level and lipid peroxidation. Second, H_2_S protected mitochondrial dysfunctions induced by rotenone, as shown by improved mitochondrial membrane potential, ATP generation, and oxygen consumption. Third, H_2_S reversed the protein expressions of GPx4, ACSL4, and Alox12, three marker proteins in ferroptosis contributing to lipid peroxidation [35,36,38]. Fourth, the blockage of ferroptosis by a selective inhibitor Fer-1 showed similar effects as H_2_S for protecting rotenone-induced lipid peroxidation, mitochondrial dysfunctions, and cell death. We also noticed that co-incubation with Fer-1 and H_2_S did not generate cumulative effect in inhibiting rotenone-induced cell death and mitochondrial dysfunctions, pointing to the possibility that alternative mechanisms other than labile iron accumulation-related ferroptosis may contribute to rotenone-induced cell death. These data demonstrated that the cardiotoxicity induced by rotenone is closely related to both apoptosis and ferroptosis, however, H_2_S would only be able to protect from ferroptotic cell death. It is not clear how rotenone induces both ferroptosis and apoptosis. Some other drugs (including oridonin and scoparone) have also been shown to trigger both ferroptosis and apoptosis [47,48]. Several reports suggest that ferroptosis may promote cellular susceptibility to apoptosis, and the damaged mitochondria and lipid peroxide from ferroptosis would initiate and drive apoptotic signals [37,49]. Single cell sequencing demonstrated that both ferroptotic and apoptotic proteins could be changed simultaneously in the same cells [50]. This evidence points to the complexity of cell fate under different disease conditions.

Different from apoptotic cell death, ferroptosis has unique features with increased membrane lipid peroxidation and occurrence of oxidative stress triggered by excessive intracellular labile iron [38]. However, the source of labile iron upon ferroptosis is unclear and debatable. In complex I, there are up to 10 enzymes containing Fe-S clusters for electron transfer and ATP generation [28,43]. It is hypothesized that the blockage of complex I by rotenone would damage these enzymes and increase labile iron level. Indeed, this current study and many others observed a lower complex I activity and a higher level of labile iron level from rotenone-treated cells [25,26]. Through the Fenton reaction, labile iron can react with H_2_O_2_ and yield a highly active hydroxyl radicals (HO•) for causing cell and tissue damage [18,23]. In contrast, by chelating intracellular iron with an iron chelator DFP, rotenone-induced cell death was suppressed. Furthermore, we observed that H_2_S mitigated rotenone-induced labile iron levels and stimulated the activity of ACO, a key Fe-S cluster-containing enzyme [7]. In consideration of this evidence, it is proposed that labile iron leaked from the disrupted Fe-S clusters may directly contribute to rotenone-induced ferroptotic cell death, and these effects would be abrogated by the gasotransmitter H_2_S.

Looking for the mechanisms on how H_2_S improves Fe-S cluster stability, we then explored the change of NFS1, a highly conserved enzyme for the very first step of Fe-S cluster synthesis by functioning as a sulfur donor [34,51]. Depletion of NFS1 leads to significant defects in mitochondrial Fe-S protein activities [39]. Although NFS1 protein was not affected by either rotenone or H_2_S, NFS1 S-sulfhydration was strikingly enhanced by exogenously applied H_2_S. S-sulfhydration is a form of protein post-translational modification, which can be induced by H_2_S for generating hydropersulfide (–SSH) in the active cysteine residues [5,15]. Hundreds of proteins have been found to be S-sulfhydated and contribute to both health and diseases [52]. By catalyzing the conversion of L-cysteine to L-alanine, NFS1 can transfer the generated sulfur to its own cysteine for forming a persulfide, which can then be transferred to the scaffold protein to form an Fe-S cluster with the aids of many other enzymes [39]. Our results suggest that H_2_S S-sulfhydration of NFS1 would facilitate Fe-S cluster synthesis, which is further supported by the results that NFS1 overexpression promotes the activity of Fe-S containing enzyme ACO only in the presence of H_2_S. It cannot be excluded that H_2_S may directly maintain the stability of Fe-S clusters in an NFS1-independent manner, which deserves further investigation. It would also be interesting to look at whether H_2_S can affect the structure and stability of other iron-containing cofactors such as heme [22].

Excessive labile iron can also be caused by an abnormal iron metabolism. To further investigate how H_2_S regulation of iron metabolism in cardiac cells under stress condition, several iron metabolism-related genes were analyzed by real-time PCR. Intracellular iron homeostasis is largely orchestrated by two iron-regulatory proteins (IRP1 and IRP2) through post-transcriptionally regulating the genes involved in iron uptake, transport, utilization, and storage [7,23]. Ferritin is the main intracellular iron storage protein, which can store up to 4500 iron atoms in a non-toxic form. TFR acts as a cellular iron importer, while FPN plays an essential role in the export of iron from cells [13]. Mitoferrin 1/2 are critical enzymes for transporting iron from cytosol into mitochondria. In contrast, ABCB7/8 are responsible for delivering iron from mitochondria to cytosol [21,53,54]. It is interestingly noted that, although *IRP1*, *IRP2*, *ferritin*, *FPN*, *ABCB7*, and *ABCB8* were all upregulated by rotenone, only *ABCB8* could be reversed by H_2_S cotreatment. Lower *ABCB8* level by H_2_S would slow down iron exit from mitochondria to cytosol. Indeed, H_2_S suppressed rotenone-induced labile iron level in cytosol to a large extent, but had less effect on labile iron level in mitochondria. Since mitoferrin 1 and 2 were not affected by either rotenone or H_2_S, it can be concluded that H_2_S inhibits iron outflow from mitochondria to cytosol by reducing *ABCB8* transcription. High levels of iron accumulated in mitochondria may facilitate NFS1-mediated Fe-S cluster synthesis. The exact mechanism by which H_2_S regulates *ABCB8* has yet to be determined.

The effects of rotenone on H_2_S signals were also evaluated in this study. With the presence of rotenone, H_2_S generation was significantly increased in cardiac cells, which may provide a compensation protection against rotenone toxicity. Surprisingly, CSE protein level and activity were not affected by rotenone, suggesting that rotenone may promote H_2_S generation independent of CSE enzyme. As it is known, iron could react with cysteine for releasing H_2_S with the help of P5P [6,7]. Given the higher level of labile iron in rotenone-incubated cells, it is possible that enhanced H_2_S generation by rotenone may be attributed to the direct interaction of iron and cysteine. Supporting this notion, iron stimulated H_2_S release from cysteine even in the absence of cell lysates, while the addition of iron chelator DFP abolished the stimulatory role of rotenone on H_2_S generation in cardiac cells.

## 5. Conclusions

**Figure 7:**
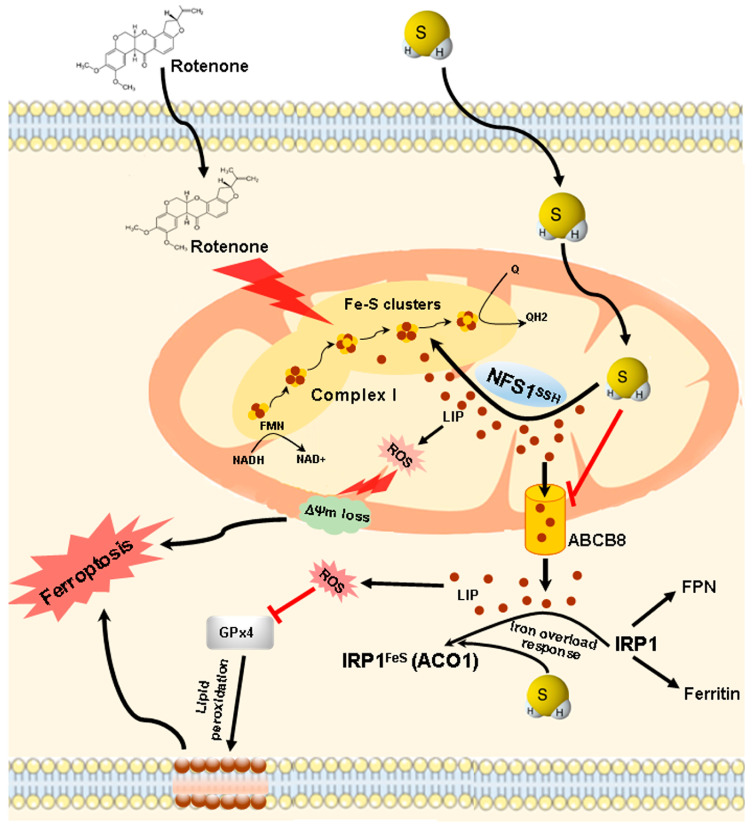
The proposed mechanisms underlying the protective roles of H_2_S against rotenone-induced ferroptosis in rat cardiac cells. Rotenone destabilizes Fe-S clusters, stimulates labile iron level and oxidative stress, and reduces GPx4 expression and mitochondrial respiratory chain complex I activity, thus causing the loss of mitochondrial membrane potential and leading to membrane lipid peroxidation, eventually inducing ferroptosis. H_2_S blocks rotenone-induced ferroptosis by inhibiting ABCB8-mediated iron efflux from mitochondria to cytosol and inducing NFS1-mediated Fe-S cluster biogenesis and stability.

In summary, this study found that rotenone destabilizes Fe-S clusters, stimulates labile iron level and oxidative stress, and damages mitochondrial functions, which eventually leads to cell death. H_2_S blocks rotenone-induced ferroptosis by inhibiting ABCB8-mediated iron efflux from mitochondria to cytosol and inducing NFS1-mediated Fe-S cluster biogenesis (Figure 7). These results indicate that H_2_S would protect cardiac cells from ischemic damage through preserving mitochondrial functions and intracellular Fe-S cluster homeostasis.

## Data Availability

Data are contained within the article.
